# Molecular Detection and Phylogenetic Analysis of Vector-Borne Pathogens from *Melophagus ovinus* in Qinghai, China

**DOI:** 10.3390/vetsci13080806

**Published:** 2026-08-14

**Authors:** Mengting Zuo, Pei Zhu, Xi Yang, Shuangshuang Zhai, Peilin Yang, Yi Zhou, Lei Tan, Jun Yao

**Affiliations:** 1 Hunan Provincial Key Laboratory of the TCM Agricultural Biogenomics, Changsha Medical University, Changsha 410219, China; zuomengting@aliyun.com; 2 College of Animal Science and Technology, Yangtze University, Jingzhou 434023, China; ngxi20030204@163.com (X.Y.); 520013@yangtzeu.edu.cn (S.Z.); 3 Yunnan Tropical and Subtropical Animal Virus Diseases Laboratory, Yunnan Animal Science and Veterinary Institute, Kunming 650224, China; zpcau@sina.com; 4 Golmud Station of Animal Husbandry and Veterinary, Haixi Autonomous Prefecture of Qinghai Province, Golmund 816000, China; woan202608@163.com

**Keywords:** *Melophagus ovinus*, vector-borne pathogens, *Bartonella melophagi*, *Rickettsia conorii*, Epidemiology, Qinghai Province

## Abstract

Vector-borne pathogens, predominantly transmitted through arthropod vectors, present a major threat to both livestock production and public health. *Melophagus ovinus* is a significant disease vector widely distributed across various pastoral regions in China. However, there has been limited research on the prevalence of vector-borne pathogens within *M. ovinus* populations in Qinghai Province, China. In this study, a total of 433 *M. ovinus* specimens were collected from Haixi and Hainan prefectures in Qinghai Province between 2024 and 2025. After identifying the parasites via morphological and molecular approaches, the specimens were grouped into 84 pools and tested for the presence of three vector-borne pathogens using PCR techniques. The findings indicated that all collected specimens exhibited morphological features consistent with *M. ovinus*, and the 18S rRNA gene sequences of the selected parasites (*n* = 7) demonstrated sequence similarities ranging from 99.1% to 100% relative to reference *M. ovinus strains*. Among the 84 *M. ovinus* pools analyzed, 68 tested positive for *Bartonella* spp. DNA; subsequent genetic characterization identified these isolates (*n* = 15) as *Bartonella melophagi*. Additionally, *Rickettsia* spp. DNA was detected in 18 pools (21.43%), genetic characterization based on the OmpA gene (*n* = 6) identified all as *Rickettsia conorii*. No DNA from *Babesia* spp. or *Theileria* spp. was detected in any of the pool. Collectively, this investigation elucidates the epidemiological characteristics of three vector-borne pathogens among *M. ovinus* populations in Qinghai, China, and provides the first evidence of a high prevalence of both *B. melophagi* and *R. conorii*, emphasizing the necessity for targeted prevention and control measures.

## Simple Summary

The present study aimed to investigate the epidemiological characteristics of *Bartonella* spp., *Rickettsia* spp., and Piroplasm in *Melophagus ovinus* populations in Qinghai Province, China. A total of 433 *M. ovinus* specimens were collected from the Haixi and Hainan prefectures and randomly divided into 84 pools for the detection of these pathogens using specific PCR methods. The results showed that 68 pools tested positive for *Bartonella* spp., and 18 pools were positive for *Rickettsia* spp. Further molecular analysis identified these pathogens as *Bartonella melophagi* and *Rickettsia conorii*, respectively. However, no Piroplasm DNA was detected in any of the pools. Overall, this study for the first time confirmed the prevalence of *B. melophagi* and *R. conorii* among *M. ovinus* specimens collected from Qinghai Province, China.

## 1. Introduction

*Melophagus ovinus*, classified within the order *Diptera* and the family *Hippoboscidae,* is an ectoparasitic species that exclusively infests the external surface of sheep [1]. *M. ovinus* acts as a hematophagous parasites on sheep, leading to anemia, weight loss, and impaired growth and development. Additionally, these parasites induce pruritus and skin inflammation, which provoke sheep to rub and bite affected areas, resulting in wool loss and skin lesions. These pathological effects significantly reduce the commercial value of wool and sheepskin products [2]. Of greater concern, *M. ovinus* functions as a vector for zoonotic pathogens, including species of Bartonella, Anaplasma, and Rickettsia, thereby posing a significant threat to both animal and human health [2,3].

*Rickettsia* spp. classified within the family *Rickettsiaceae* and the order *Rickettsiales*, are intracellular parasitic Gram-negative bacteria that can infect a variety of mammalian hosts, including humans, and are associated with diverse clinical manifestations [4,5]. In China, Rickettsia strains transmitted by *M. ovinus* include *R. massiliae*, *R. sibirica*, *R. conorii*, and *R. parkeri * [6,7,8]. *Bartonella* spp. belong to a genus of Gram-negative, aerobic bacteria that can infect various mammalian species, including goat, cattle, rodents, and humans [9]. In humans, *Bartonella* spp. infections can result in diseases including cat scratch disease, trench fever, oroya fever, and sometimes severe neurological disorders, with clinical presentations largely dependent on the specific species of the pathogen involved [10]. Several *Bartonella* species have been identified in these hosts, including *B. grahamii*, *B. elizabethae*, and *B. tribocorum*, etc. [11]. Piroplasms consist of the genera Babesia and Theileria, which infect red blood cells and vascular endothelial cells in susceptible animals, leading to clinical signs such as high fever, anemia, and swollen lymph nodes [12,13].

**Figure 1:**
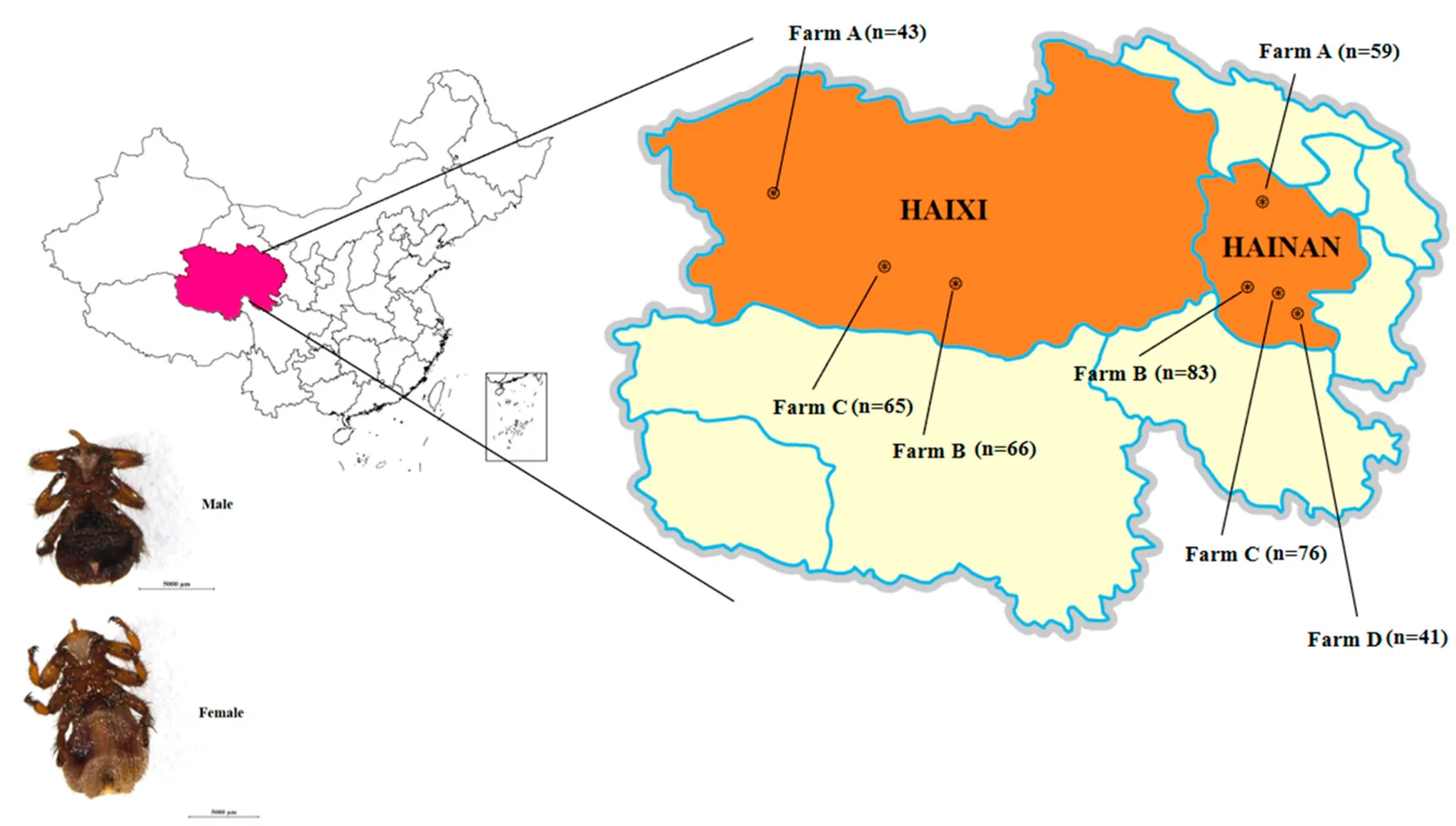
Sample collection sites of *M. ovinus* in Qinghai Province of China.

Qinghai Province, situated in western China, occupies a prominent position in the northeastern sector of the Qinghai–Tibet Plateau (Figure 1). This region is characterized by a highly developed livestock farming industry; however, the widespread occurrence of vector-borne diseases impedes the sustainable growth of this industry which are mainly transmitted by arthropod, including ticks and *M. ovinus*. A variety of vector-borne pathogens, including *Rickettsia* spp., *Theileria* spp., and *Brucella* spp., have been confirmed as prevalent within tick populations in Qinghai Province [14,15,16]. However, comprehensive investigations into the vector-borne pathogens transmitted specifically by *M. ovinus* populations in this region remain limited. Only Zhang et al. shown that *Anaplasma ovis*, *A, bovis*, *A. phagocytophilum*, and *T. ovis*, but not *Babesia* spp., *Rickettsia* spp., and *Borrelia* spp. were found in *M. ovinus* populations in Qinghai Province [4]. However, whether *Rickettsia* spp. and *Brucella* spp. are prevalent in *M. ovinus* species remains to be explored. In this study, a total of 433 *M. ovinus* samples were collected from Qinghai Province during the period of 2024 to 2025. Species identification was conducted through morphological analysis and 18S rRNA sequencing. Genomic DNA extracted from the *M. ovinus* samples was subjected to PCR assays targeting *Bartonella* spp., *Rickettsia* spp., and Piroplasms to assess the prevalence and species diversity of these pathogens.

## 2. Materials and Methods

### 2.1. Sample Collection and Morphological Identification

A total of 433 *M. ovinus* isolates were collected from the surface of Tibetan sheep in selected pastoral regions of Haixi and Hainan Prefectures, Qinghai Province, during the period from June to September in 2024 and 2025. Of these, 174 specimens were obtained from three pastures in Haixi Prefecture, while 259 were collected from four sheep farms in Hainan Prefecture (Figure 1). These collected samples were cleansed of surface dust using clean water, subsequently placed in sterile 50 mL centrifuge tubes with 75% ethanol solution for preservation. Next, these samples were transported under low temperature conditions to Yangtze University. Individual *M. ovinus* specimen was rinsed with sterile distilled water to remove residual contaminants, surface moisture was absorbed, and specimens were observed under a stereomicroscope. Detailed morphological features, including reproductive pores, valve plates, setae bearing limbs, and the anal sulcus, were carefully examined and photographed. The sex and developmental stage were identified following the methodology established in a previous study [17].

### 2.2. DNA Genome Extraction and Molecular Identification

These *M. ovinus* samples were repeatedly rinsed with sterile distilled water to remove residual surface alcohol. The specimen was individually transferred to a mortar, and a small quantity of liquid nitrogen was introduced prior to comprehensive grinding. Approximately 30 mg of the homogenized sample was collected for genomic DNA extraction using the TIANamp Blood DNA Kit (Tiangen Biotech, Beijing, China) following the manufacturer’s instructions. The extracted DNA genome was dissolved in 50 μL of Tris-EDTA buffer and stored at −20 °C for preservation.

For the molecular identification of *M. ovinus*, targeted PCR assays were performed to amplify the 18S rRNA gene utilizing one pair of specific primer, as detailed in Table 1. In brief, PCR volume (50 μL) contained of 25 μL of 2 × Rapid Taq Master Mix (Vazyme Biotech, Nanjing, China), 1.0 μL of each primer (10 µM), 2.0 μL of template DNA, and 21 μL of RNA-free water. The amplification conditions were set as follows: 94 °C for 5 min; 35 cycles of 94 °C for 30 s, 55 °C for 30 s, and 72 °C for 30 s; followed by 72 °C for 7 min. Following gel electrophoresis, the representative positive PCR products (n = 7) were purified and subsequently submitted to BGI Genomics (Shenzhen, China) for sequencing.

### 2.3. Species-Specific Pcr for Detecting Vector-Borne Pathogens

In this study, all 433 *M. ovinus* samples were randomly grouped into 84 pools with each pool containing 4 to 6 samples (Table 2). To guarantee that each pool corresponded to a single epidemiological unit, specimens collected from the same location and on the same date were grouped together. Pooling was performed randomly only within these groups, not between different sites or collection dates. For each pool, specimens were combined and grouped together in liquid nitrogen, and approximately 30 mg of the homogenized powder was used for genomic DNA extraction following the same procedures as described in Section 2.2. Subsequently, targeted PCR assays were conducted to detect the presence of DNA from three vector-borne pathogens in the collected *M. ovinus* specimens. As detailed in Table 1, these PCR protocols specifically amplified the *OmpA* gene for *Rickettsia* spp., the *gltA* gene for *Bartonella* spp., and the *18S rRNA* gene for Piroplasma, respectively. In brief, the PCR system and reaction conditions were consistent with those targeting *M. ovinus* genome, with the exception of the annealing temperature, which varied for certain pathogens (Table 1). Following gel electrophoresis, the representative positive PCR products were purified and subsequently submitted to BGI Genomics (Shenzhen, China) for sequencing.

### 2.4. Bioinformatics Analysis

The nucleotide sequences of these representative strains were retrieved from the GenBank database. Comparative genetic analyses between the acquired sequences and their respective reference sequences were performed utilizing DNAStar software version 7.10. Phylogenetic trees were constructed using MEGA version 11.0 software, utilizing the Neighbor-Joining (NJ) method as described in prior research [22].

### 2.5. Statistics Analysis

The combined detection rate and its associated 95% confidence intervals (95% CI) for the three vector-borne pathogens analyzed in this study were calculated utilizing the epidemiological calculator EpiTools. The influence of multiple potential risk factors, such as geographic location, gender, and breeding system, on the pooled detection rates of these pathogens was evaluated using the Chi-square test implemented in SPSS version 24.0 (SPSS Inc., Chicago, IL, USA). Statistical significance was established at a threshold of *p* < 0.05.

## 3. Results

### 3.1. Morphological and Molecular Identification of M. ovinus

**Figure 2:**
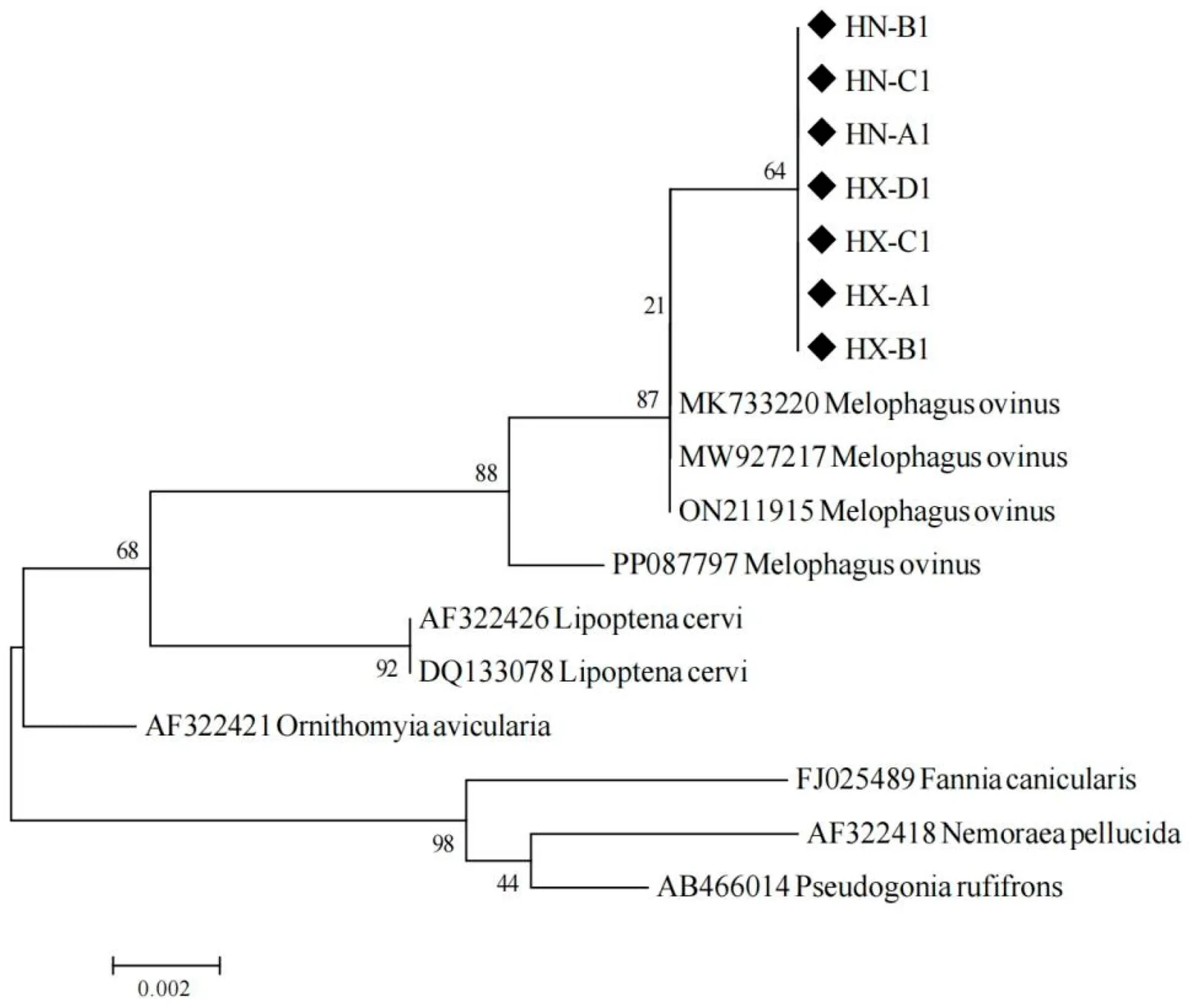
Phylogenetic tree based on the 18S rRNA gene sequence of *M. ovinus* strains collected in this study and reference strains was constructed using the NJ method based on the Kimura 2-parameter model, implemented in version 11.0 software. The robustness of the tree topology was evaluated through a bootstrap analysis with 1000 replicates.

A total of 433 sheep keds were obtained from the exterior of seven sheep flocks located in Haixi Prefecture (n = 3) and Hainan Prefecture (n = 4) within Qinghai Province of China. Based on their morphological features, all specimens were identified as *M. ovinus*, comprising 187 males and 246 females (Figure 1). Furthermore, seven representative specimens (GenBank accession no. PZ728753–PZ728759) were randomly chosen from seven distinct flocks for subsequent molecular identification. The amplified 18S rRNA gene sequences obtained from the genomes of these specimens measured 575–578 bp in length. Sequence similarity analysis revealed that these strains exhibited a nucleotide sequence similarity ranging from 99.1% to 99.8% when compared with *M. ovinus* strains deposited in the GenBank database, while they showed reduced sequence identity (<95.8%) relative to other reference strains. Additional phylogenetic analysis classified them as *M. ovinus,* since these strains clustered together with *M. ovinus* strains from the GenBank database into a single clade, which was distinctly separated from other parasites (Figure 2).

### 3.2. Molecular Detection Results of Three Vector-Borne Pathogen Species

As shown in Table 2, molecular analysis revealed that 74 pools tested positive for the presence of at least one vector-borne pathogen, corresponding to an average pooled detection rate of 88.10% (74/84, 95%CI: 81.18–95.02). Moreover, 68 (80.95%, 95%CI: 72.95–89.35) pools from all investigated farms were positive for *Bartonella* spp. While only 18 (21.43%, 95%CI: 12.65–30.21) pools from two farms tested positive for *Rickettsia* spp. Co-infection with *Bartonella* spp. and *Rickettsia* spp. were only detected in 8 pools. However, the DNA of *Piroplasma* species was not detected in any of the pools.

Next, we examined the influence of potential risk factors, such as geographic region, gender, and breeding type, on the pooled detection rates of vector-borne pathogens in *M. ovinus* specimens collected from Qinghai Province. The results indicated that *Rickettsia* spp. infection was exclusively identified in Haixi Prefecture, with no cases detected in Hainan Prefecture. Conversely, the detection rates of *Bartonella* spp. infection did not exhibit a statistically significant difference between the two regions. The pooled detection rates of these two pathogens did not exhibit significant differences between male and female subjects (*p* > 0.05); however, their pooled prevalence was markedly higher in free-range farms compared to large-scale farming operations (*p* < 0.05).

### 3.3. Genetic Diversity and Phylogenetic Characterization of Bartonella Species Strains

**Figure 3:**
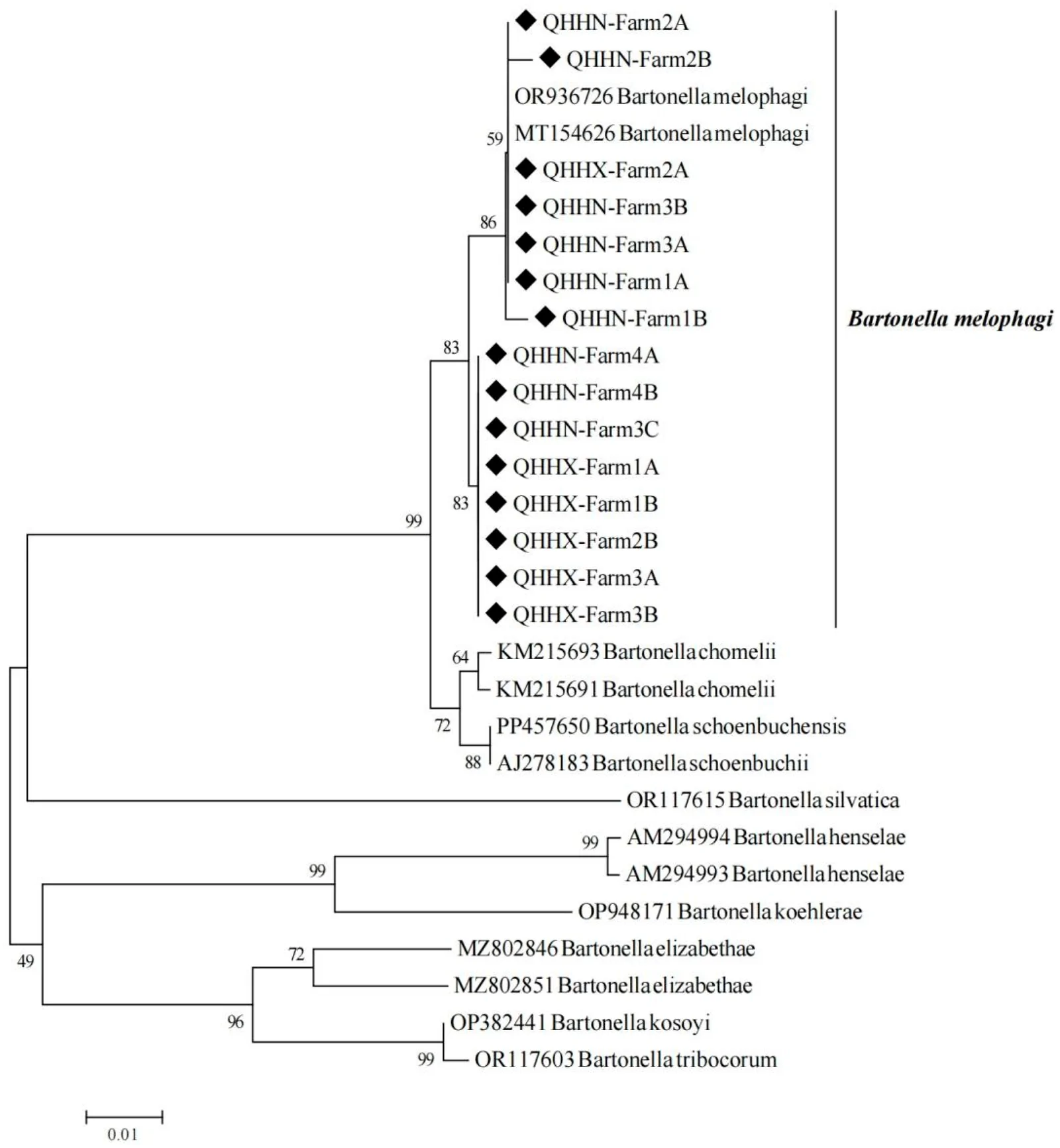
Phylogenetic tree based on the gltA gene sequence of *B. melophagi* strains collected in this study and reference strains was constructed using the NJ method based on the Kimura 2-parameter model, implemented in version 11.0 software. The robustness of the tree topology was evaluated through a bootstrap analysis with 1000 replicates.

To further explore the genetic characteristics of the identified *Bartonella* spp. strains, *gltA* gene sequences were successfully obtained from 15 *Bartonella* spp. strains, each comprising 327 bp in length (GenBank accession no. PZ740155–PZ740169). Notably, These sequences showed a nucleotide similarity of 99.1% to 100% among themselves, and displayed 99.4% to 100% similarity when compared to the reference *Bartonella melophagi* strains (GenBank accession no. MT154626 and OR936726). In contrast, sequence similarity with other *Bartonella* spp. strains, including *B. chomelii*, *B. henselae*, *B. wilsonii*, and others, ranged from 85.0% to 98.5%. Moreover, a phylogenetic tree was generated utilizing the NJ method implemented in MEGA 11.0 version software, using the *gltA* gene sequences of *Bartonella* spp. strains. As illustrated in Figure 3, all *Bartonella* spp. strains isolated in this study clustered within a single clade alongside the *B. melophagi* reference strains obtained from the GenBank database. This clade was clearly separate from the branches linked to other *Bartonella* species due to its distinct clustering.

### 3.4. Genetic Diversity and Phylogenetic Characterization of Rickettsia Species Strains

**Figure 4:**
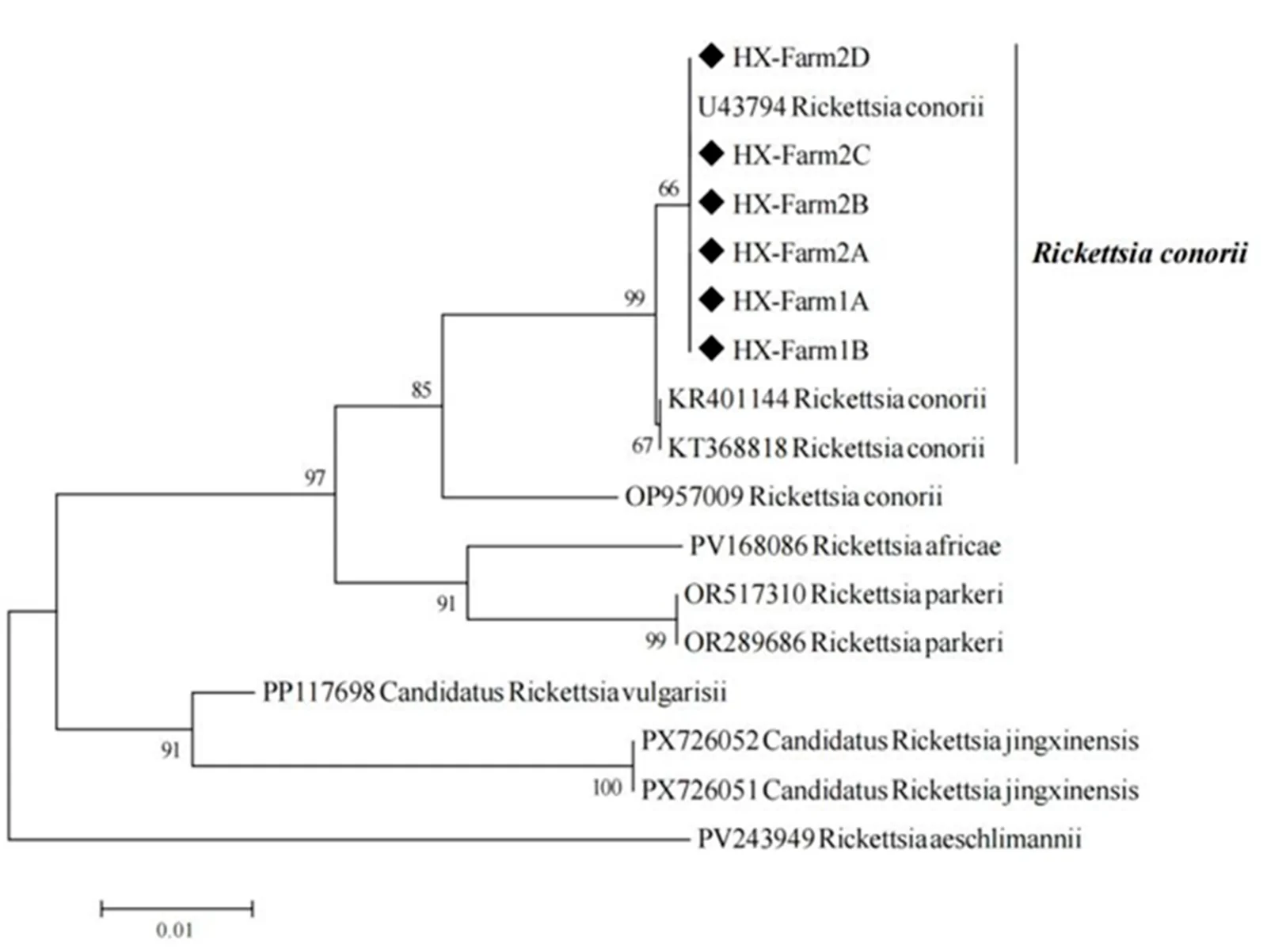
Phylogenetic tree based on the OmpA gene sequence of *R. conorii* strains collected in this study and reference strains was constructed using the NJ method based on the Kimura 2-parameter model, implemented in version 11.0 software. The robustness of the tree topology was evaluated through a bootstrap analysis with 1000 replicates.

A total of six OmpA gene sequences, each comprising 569 bp, were successfully sequenced from *Rickettsia* spp. strains in this study (GenBank accession no. PZ737684–PZ737689). Remarkably, these sequences demonstrated complete nucleotide identity with a reference *Rickettsia conorii* strain (GenBank accession no. U43794), and shared 96.4% to 98.4% sequence identity with other *R. conorii* strains. In contrast, they displayed lower sequence similarity (less than 96.0%) when compared to strains of other *Rickettsia* species. Similarly, the six Rickettsia strains identified in this study were clustered together with the reference *R. conorii* strains, and were distinctly separated from the branches of other *Rickettsia* species (Figure 4).

## 4. Discussion

*M. ovinus* has been prevalent in regions such as Xizang, Xinjiang, Qinghai, Inner Mongolia and Gansu across China, significantly impeding the advancement of the country’s sheep industry [1,19,20]. Although there have been no documented cases of *M. ovinus* biting humans, the diverse array of pathogens which harbor presents potential threats to human health [21]. In previous research, *M. ovinus* has been documented to carry *Anaplasma* species in Qinghai Province [3], as well as *Rickettsia* and *Anaplasma* species in Xinjiang [8].

*Rickettsia* spp. constitute a prevalent zoonotic pathogen and are acknowledged as important agents of vector-borne infectious diseases. This genus can be harbored by both *M. ovinus* and ticks, and is widely distributed across China, including in Qinghai Province [16]. However, a prior investigation reported the absence of *Rickettsia* spp. positive cases detected in *M. ovinus* samples collected from Qinghai Province [3]. The present investigation verified the prevalence of *Rickettsia* spp. within *M. ovinus* populations in Qinghai Province. The overall pooled positive rate of this genus was determined to be 21.43% (18 out of 84 pools), while which was only detected in Haixi Prefecture. Subsequent genetic analysis targeting the OmpA gene of *Rickettsia* spp. showed that all sequenced samples (n = 6) were identified as *R. conorii.* Previous study has documented that the prevalence of *R. principis*, *R. jingxinensis*, *R. raoultii*, *R. barbariae*, and *R. mamionii* have been detected in tick samples collected from Qinghai Province [22]. However, the occurrence of *R. conorii* in this region has not been previously reported. It is noteworthy that *R. conorii* has been detected in several Chinese provinces, including Xinjiang, Yunnan, and Shandong [23], and the clinical symptoms of *R. conorii* infection in human are characterized by fever, rash, and flu-like manifestations [23]. In this context, there is an urgent need for additional research to elucidate the transmission dynamics and public health implications of *R. conorii* in Qinghai. Furthermore, experimental investigations into vector competence and pathogenicity are essential for evaluating the potential risks posed by emerging rickettsial infections in this area.

*Bartonella* spp. are implicated in a range of infectious diseases, including cat scratch disease, endocarditis, trench fever, and Carrion’s disease [11]. In the current study, *Bartonella* spp. were identified in *M. ovinus* samples collected from Qinghai Province, exhibiting an average pooled positivity of 80.95% (68 out of 84 pools). A recent investigation reported a 100% (214/214) prevalence of *Bartonella* spp. in *M. ovinus* specimens from Xinjiang [1]. These results collectively suggest that *Bartonella* spp. are extensively distributed within *M. ovinus* populations across various regions of China, which may due to the symbiotic relationship between *Bartonella* spp. and *M. ovinus* [24]. Further genetic analysis focusing on the gltA gene revealed that all 15 *Bartonella* spp. isolates in this study were identified as *B. melophagi*, indicating that *B. melophag* was the most common in the studied areas. The high prevalence of *B. melophag* in *M. ovinus* has been documented in other regions, including Poland (88.63%, 694/783) [25], the Czech Republic (100.0%) [26], and China (100.0%, 17/17) [27]. Moreover, *B. melophagi* has been identified in yaks within Qinghai Province, with a pooled positive rate of 30.1% (246/818) [28]. When considered alongside previously documented human cases manifesting cardiovascular symptoms [29], these data collectively highlight that *B. melophagi* is not simply an incidental animal-associated bacterium. Instead, it represents a broadly distributed and unrecognized zoonotic pathogen with significant implications for public health.

It is important to note that no Piroplasma DNA was detected in any of the pools, which does not necessarily indicate that this species is absent from *M. ovinus* specimens or goats in the studied areas. Piroplasma (*Theileria ovis*) has been identified in *M. ovinus* samples collected from Qinghai Province [3]. And various *Theileria* species are commonly found in goat populations across different regions of Qinghai Province [30]. These negative results probably stem from the limited sampling range and small sample size. Future research should increase the sampling regions and number of samples, as well as use more sensitive molecular tests to better determine the local prevalence.

This study presents several limitations. Geographically, the sampling was confined to only two prefectures within Qinghai Province, which may restrict the applicability of the results to other regions. From a methodological perspective, the use of pooled specimens precluded the determination of individual infection rates and may have led to an underestimation of pathogen prevalence. Furthermore, the PCR-based detection method identified pathogen DNA exclusively, without accompanying serological confirmation or bacterial isolation, thereby precluding differentiation between active infections and the presence of non-viable DNA or contamination. Crucially, the lack of paired host blood samples and vector competence assays renders the inferred role of *M. ovinus* in pathogen transmission suggestive rather than definitive. Future research should incorporate more extensive geographic sampling, individual specimen analysis, and experimental transmission studies to robustly establish the vectorial capacity of *M. ovinus* and its implications for public health.

## 5. Conclusions

This study presents the first comprehensive evidence of the prevalence and genetic characterization of *B. melophagi* and *R. conorii* in *M. ovinus* specimens collected from Qinghai Province, China. The elevated detection rates of these pathogens suggest that *M. ovinus* may be involved in the transmission of these zoonotic bacterial agents within this geographic area. The results offer critical baseline information for evaluating potential disease risks and underscore the imperative for continuous monitoring and the development of effective intervention strategies to protect both animal and human health in pastoral environments.

## Figures and Tables

**Table 1:** Detailed information of the primers used in this study, including their target genes, sequence details, PCR product sizes, purposes, and references.

Primer	Sequence 5′-3′	Length	Annealing Temperature	Purpose	Reference
18S rRNAF	AACTTGTGCTTCATACGGG	520 bp	55 °C	Molecular identification of *Melophagus ovinus*	[18]
18S rRNAR	GCGACTGAGAGAGCCATAA				
BETT-gltAF	GGGGACCAGCTCATGGTGG	379 bp	56 °C	Molecular detection of *Bartonella* spp.,	[19]
BETT-gltAR	AATGCAAAAAGAACAGTAAACA				
LKCT-OmpAF	ATGGCGAATATTTCTCCAAAA	631 bp	55 °C	Molecular detection of *Rickettsia* spp.,	[20]
LKCT-OmpAR	GTTCCGTTAATGGCAGCATCT				
18S-rRNAF	GTCTTGTAATTGGAATGATGG	450 bp	55 °C	Molecular detection of Piroplasma	[21]
18S-rRNAR	TAGTTTATGGTTAGGACTACG				

**Table 2:** Pooled positive rate of *Bartonella* spp. and *Rickettsia* spp. among *M. ovinus* specimens collected from Qinghai Province.

		Number of Sample	Number of Pooled	Number of Positive Pooled (n)/Positive Rate			
				*Bartonella* spp.	*p*-Value	*Rickettsia* spp.	*p*-Value
Region	Haixi	174	31	27/87.09%	0.273	12/38.71%	Not calculated
	Hainan	259	53	41/77.36%		0/0	
Gender	Female	246	49	40/81.63%	0.851	10/20.41%	0.899
	Male	187	35	28/80.0%		8/22.86%	
Breeding type	Large scale	340	64	48/75.0%	<0.05	7/10.94%	<0.05
	Small scale	93	20	20/100.0%		11/55.0%	
	In total	433	84	68/80.95%	-	18/21.43%	-

## Data Availability

The original contributions presented in this study are included in the article. Further inquiries can be directed to the corresponding authors.

## References

[1] Huang K., Zhang X., Xiong N., Sun L., Zhao X., Zhou K., Wu J. (2024). First metagenomic sequencing for the analysis of microbial community populations of adults and pupae of *Melophagus ovinus* in Xinjiang, China. Front. Vet. Sci..

[2] Liu Y., Ma Y., Tian H., Yang B., Han W., Zhao W., Chai H., Zhang Z., Wang L., Chen L. (2022). First determination of DNA virus and some additional bacteria from *Melophagus ovinus* (sheep ked) in Tibet, China. Front. Microbiol..

[3] Zhang Q., Wang Y., Li Y., Han S., Wang B., Yuan G., Zhang P., Yang Z., Wang S., Chen J. (2021). Vector-Borne Pathogens with Veterinary and Public Health Significance in *Melophagus ovinus* (Sheep Ked) from the Qinghai-Tibet Plateau. Pathogens.

[4] Walker D.H., Ismail N. (2008). Emerging and re-emerging rickettsioses: Endothelial cell infection and early disease events. Nat. Rev. Microbiol..

[5] Ly N., Guzman A.A., Engström P., Burke T.P. (2026). Innate immune responses to Rickettsia: Emerging themes and contrasts between species. Curr. Opin. Microbiol..

[6] Liu D., Wang Y., Zhang H., Liu Z., Wureli H., Wang S., Tu C., Chen C. (2016). First report of Rickettsia raoultii and R. slovaca in *Melophagus ovinus*, the sheep ked. Parasit. Vectors.

[7] Liu G., Zhao S., Tan W., Hornok S., Yuan W., Mi L., Wang S., Liu Z., Zhang Y., Hazihan W. (2021). Rickettsiae in red fox (*Vulpes vulpes*), marbled polecat (*Vormela peregusna*) and their ticks in northwestern China. Parasit. Vectors.

[8] Li S., Zhang L., Li Z., Song H., Que Z., Zhao S., Li Y., Guo Y., Wu J. (2023). Detection of *Rickettsia* spp. and *Anaplasma ovis* in *Melophagus ovinus* from southern Xinjiang, China. Med. Vet. Entomol..

[9] Jones B.P., Wawman D.C., Johnson N. (2026). Detection of *Bartonella schoenbuchensis* and a novel sigmavirus within the microbiome of deer keds (*Lipoptena cervi*) from the United Kingdom. Parasit. Vectors.

[10] Billeter S.A. (2022). A Review of Bartonella Infections in California-Implications for Public and Veterinary Health. J. Med. Entomol..

[11] Krügel M., Król N., Kempf V.A.J., Pfeffer M., Obiegala A. (2022). Emerging rodent-associated Bartonella: A threat for human health?. Parasit. Vectors.

[12] Woods K., Perry C., Brühlmann F., Olias P. (2021). Theileria’s Strategies and Effector Mechanisms for Host Cell Transformation: From Invasion to Immortalization. Front. Cell Dev. Biol..

[13] Schnittger L., Ganzinelli S., Bhoora R., Omondi D., Nijhof A.M., Florin-Christensen M. (2022). The Piroplasmida Babesia, Cytauxzoon, and Theileria in farm and companion animals: Species compilation, molecular phylogeny, and evolutionary insights. Parasitol. Res..

[14] Chen Q., Li Z., Kang M., Hu G., Cai J., Li J., Han X., Chen C., He S., Hu X. (2024). Molecular identification of tick (Acari: Ixodidae) and tick-borne pathogens from Przewalski’s gazelle (Procapra Przewalskii) and Tibetan sheep (*Ovis aries*) in Qinghai Lake National Nature Reserve, China. Heliyon.

[15] Han Y., Li Z., Fu Y., Liu J., Jia D., Li C., Yin H., Lei M. (2026). Genetic diversity of Theileria species identified from questing and parasitic ticks in selected areas in Qinghai, China. Vet. Res. Commun..

[16] Zhang D., Ma Y., Zhao X., Yang H., Li X., Wang G., Hu Y., Tang S., Li R., Li S. (2026). Molecular Detection and Identification of Bacterial Pathogens in Qinghai Province, China. Pathogens.

[17] Kumsa B., Socolovschi C., Parola P., Rolain J., Raoult D. (2012). Molecular detection of Acinetobacter species in lice and keds of domestic animals in Oromia Regional State, Ethiopia. PLoS ONE.

[18] Duan D.Y., Wang Y.Q., Chen T.Y. (2017). Morphological and Molecular Identification of *Melophagus ovinus*. Acta Vet. Zootech. Sin..

[19] Maurin M., Raoult D. (1996). Bartonella (Rochalimaea) quintana infections. Clin. Microbiol. Rev..

[20] Roux V., Fournier P.E., Raoult D. (1996). Differentiation of spotted fever group rickettsiae by sequencing and analysis of restriction fragment length polymorphism of PCR-amplified DNA of the gene encoding the protein rOmpA. J. Clin. Microbiol..

[21] Vascellari M., Ravagnan S., Carminato A., Cazzin S., Carli E., Da Rold G., Lucchese L., Natale A., Otranto D., Capelli G. (2016). Exposure to vector-borne pathogens in candidate blood donor and free-roaming dogs of northeast Italy. Parasit. Vectors.

[22] Duan Y., Liu N., Tan L., Gao L., Zhu G., Tao Z., Liu B., Wang A., Yao J. (2025). Isolation and biological characterization of a variant pseudorabies virus strain from goats in Yunnan Province, China. Vet. Res. Commun..

[23] Tang J., Li F., Cheng T., Duan D., Liu G. (2018). Comparative analyses of the mitochondrial genome of the sheep ked *Melophagus ovinus* (Diptera: Hippoboscidae) from different geographical origins in China. Parasitol. Res..

[24] Li L., Xie H., Li Z., Tang W., Zhang C., Qi X., Luo R., Chui W., Kui J., Huang F. (2026). Complete Mitochondrial Genome of *Melophagus ovinus* from Qinghai-Tibet Plateau Provides Evidence for D-Loop Length Polymorphism. Genes.

[25] Werszko J., Asman M., Witecka J., Steiner-Bogdaszewska Ż., Szewczyk T., Kuryło G., Wilamowski K., Karbowiak G. (2021). The role of sheep ked (*Melophagus ovinus*) as potential vector of protozoa and bacterial pathogens. Sci. Rep..

[26] Luo C., Song Y., Xia L., Liu M., Feng H., Xiao L., Xu M., Cai X., Cui J., Xiang R. (2024). Molecular epidemiological study on tick-borne pathogens in Qinghai Province, Northwestern China. Biosaf. Health.

[27] Xu N., Liu H., Qu C., Wen S., Zou W., Chang C., Wang G. (2023). The presence of foci of *Rickettsia conorii* infection in China. Infect. Med..

[28] Halos L., Jamal T., Maillard R., Girard B., Guillot J., Chomel B., Vayssier-Taussat M., Boulouis H.J. (2004). Role of Hippoboscidae flies as potential vectors of *Bartonella* spp. infecting wild and domestic ruminants. Appl. Env. Microbiol..

[29] Kumsa B., Parola P., Raoult D., Socolovschi C. (2014). Bartonella melophagi in *Melophagus ovinus* (sheep ked) collected from sheep in northern Oromia, Ethiopia. Comp. Immunol. Microbiol. Infect. Dis..

[30] Rudolf I., Betášová L., Bischof V., Venclíková K., Blažejová H., Mendel J., Hubálek Z., Kosoy M. (2016). Molecular survey of arthropod-borne pathogens in sheep keds (*Melophagus ovinus*), Central Europe. Parasitol. Res..

